# Development and Initial Testing of a Personalized, Adaptive, and Socially Focused Web Tool to Support Physical Activity Among Women in Midlife: Multidisciplinary and User-Centered Design Approach

**DOI:** 10.2196/36280

**Published:** 2022-07-26

**Authors:** Danielle Arigo, Andrea F Lobo, M Cole Ainsworth, Kiri Baga, Kristen Pasko

**Affiliations:** 1 Department of Psychology Rowan University Glassboro, NJ United States; 2 Department of Family Medicine Rowan School of Osteopathic Medicine Stratford, NJ United States; 3 Department of Computer Science Rowan University Glassboro, NJ United States

**Keywords:** user-centered design, digital health, eHealth, women’s health, midlife, physical activity, social support, social comparison, mobile phone

## Abstract

**Background:**

Women in midlife are vulnerable to developing cardiovascular disease, particularly those who have conditions such as hypertension. Physical activity (PA) can reduce risk, but efforts to promote PA in this population have been only modestly effective. More attention to social influences on PA behavior may be useful, particularly social support and social comparison processes. Activating these processes with digital tools can provide easy access that is flexible to the needs of women in midlife.

**Objective:**

This paper describes the user-centered design processes of developing and conducting initial evaluation of a personalized and adaptive web application, tailored to the social needs of women in midlife. The goal was to gather feedback from the population of interest, before and during the design process.

**Methods:**

This study was conducted in 4 stages. The first and second authors (DA and AFL) developed technical specifications, informed by their experience with the population of interest. We collected feedback on potential content for the web application with women in midlife using both interviews (5/10, 50%; mean age 47.4, SD 6.66 years; mean BMI 35.3, SD 9.55 kg/m^2^) and surveys (5/10, 50%; mean age 51, SD 6.60 years; mean BMI 32.7, SD 8.39 kg/m^2^). We used their feedback to inform support messages and peer profiles (ie, sources of social comparison information). Nine members of the behavioral science team and 3 testers unfamiliar with the web application completed internal testing. We conducted naturalistic functionality testing with a different group of women in midlife (n=5; mean age 50, SD 6.26 years; mean BMI 30.1, SD 5.83 kg/m^2^), who used the web application as intended for 4 days and provided feedback at the end of this period.

**Results:**

Iterative storyboard development resulted in programming specifications for a prototype of the web application. We used content feedback to select and refine the support messages and peer profiles to be added. The following 2 rounds of internal testing identified bugs and other problems regarding the web application’s functioning and full data collection procedure. Problems were addressed or logged for future consideration. Naturalistic functionality testing revealed minimal further problems; findings showed preliminary acceptability of the web application and suggested that women may select different social content across days.

**Conclusions:**

A multidisciplinary and user-centered design approach led to a personalized and adaptive web application, tailored to the social needs of women in midlife. Findings from testing with this population demonstrated the feasibility and acceptability of the new application and supported further development toward its use in daily life. We describe several potential uses of the web application and next steps for its development. We also discuss the lessons learned and offer recommendations for future collaborations between behavioral and computer scientists to develop similar tools.

## Introduction

### Background

Cardiovascular disease (CVD) remains the leading cause of mortality worldwide, and its public health burden continues to grow [1-3]. Risk for CVD increases with age, with the increased prevalence of risk conditions such as type 2 diabetes and hypertension [4]. Women in midlife (aged 40-60 years [5]) with these conditions are particularly vulnerable; their risk is already heightened by the processes of aging [6], associated weight gain [7] and menopause transition [4,8]. Engaging in regular physical activity (PA) substantially reduces their risk for CVD [9] and has many other physical and mental health benefits for women [10]. However, the gender disparity in PA engagement that favors men across the life span widens during midlife [11], and less than half of the women in the United States in this age range meet US national PA guidelines [12]. Consequently, promoting PA among women in midlife with elevated CVD risk has received considerable empirical and clinical attention as a key pathway for improving cardiovascular health in this population [4,13].

Behavior change techniques such as goal setting, intention formation, and self-monitoring show positive effects on PA adoption and maintenance, at least in the short term (ie, 6 months to 1 year [14,15]). However, these techniques are not widely effective for increasing PA among women in midlife [16]. In addition to widespread challenges such as low motivation [17], many women in midlife face barriers to prioritizing their own health in daily life. Women in this group often serve a variety of caretaking roles in addition to other personal and professional responsibilities [18], and many perceive that taking time for their own PA is selfish [19]. Thus, there is a clear need for novel, low-burden PA resources that are tailored specifically for this population. This paper describes a user-centered design (UCD) approach to address the PA needs and preferences of women in midlife, with a tailored digital tool.

### Social Influences on PA Among Women in Midlife

Great attention to *social influences on PA* in interventions may be particularly useful for women in midlife, as women in this age range often cite lack of social support for PA as a key barrier to engagement [19]. Social support for behavior change is a multifaceted construct that involves various types of behavior or communication from others. For example, informational support involves providing resources or suggestions (eg, reasons or ways to change a behavior), whereas emotional support involves demonstrating compassion (eg, encouragement or validation). Individuals differ in the type or types of support they prefer and find helpful [20], and their preferences and responses may fluctuate across days or weeks (eg, based on mood or perceived barriers [21]). A simple way to provide support in digital interventions for health behavior change is via text-based messages, which can be assigned by the system or self-selected by users to meet their immediate needs. Common categories of support in such interventions include information (ie, advice and tips for ways to change health behaviors), encouragement (ie, motivational messages to reinforce and promote health behavior efforts or success), and accountability (ie, tips for increasing commitment to health behavior goals or plans [22]).

Similarly, social comparisons, or self-evaluations relative to others [23], show influence on PA among women in midlife. This group expresses desire for and positive subjective response to PA role models, particularly those who are similar to them (eg, regarding demographic background, caregiving responsibilities, and workload [24,25]). A range of PA-based comparisons can have positive effects. Comparing one’s own PA behavior with that of someone who engages in more PA (eg, steps or minutes of aerobic exercise per day), or *upward comparison*, can provide inspiration, motivation, and guidance to achieve a similar outcome [26]. Comparing one’s own PA behavior with that of someone with the same level of PA engagement, or *lateral comparison*, can provide assurance of *keeping up* to a relevant standard [27]. Comparing one’s own PA behavior with that of someone who engages in less PA, or *downward comparison*, can prompt satisfaction with one’s own PA behavior and motivation to continue PA efforts to stay ahead of others [28]. Importantly, both upward and downward comparisons can have the opposite effects: upward comparisons can lead to dejection and disappointment (particularly when there is a very large or consistent discrepancy between one’s own and the target’s PA [29,30]), and downward comparisons can confirm that one’s own PA situation is bad (or likely to become so [31]), and lead to decreased PA. Lateral comparisons are least likely to prompt negative responses; however, the range of their effects is currently unclear. The consequences of all 3 types of comparisons for PA also differ between people and fluctuate within the same person over time [32-36].

Some existing studies have attempted to activate social support and comparison processes during group interventions to promote PA among women in midlife [37,38]. Others have matched intervention participants with one another to create PA partnerships that foster support and comparison, with matching based on demographic characteristics [39,40]. Findings from these studies suggest that women in midlife will engage in discussions that offer mutual support for PA between participants and will respond to social comparison opportunities (eg, by indicating that they are motivated to keep up with others in the program or use them as role models). Importantly, however, a subset of these studies show that women differ in their responses to opportunities for support or comparison and that these opportunities can result in decreased PA motivation or behavior [39,40]. A reason for this heterogeneity in response is that existing approaches do little to ensure that opportunities for support or comparison are those that match individual preferences. Furthermore, users’ preferences may not be most effective for fostering PA adoption or maintenance [41]. In addition to facilitating on-demand PA resources, digital tools that allow self-selection of support and comparison prompts can provide useful information about alignment between preferences and needs and inform improvements to intervention approaches (for women in midlife and more broadly).

### Digital PA Support: Personalization, Adaptation, and UCD

Historically, evidence-based interventions to promote health behaviors such as PA have been fixed, such that all participants receive the same intervention content over time [42]. Digital tools have the potential to account for individual differences in participants’ responses to intervention content. *Personalization*, or the use of specific content for its alignment with individual characteristics of the user, is intended to increase users’ attention by signaling that information is meant specifically for them. Personalized content is expected to be more relevant than generic content, and thus, increase the power of communication [43]. Common personalization strategies in digital PA interventions include individualized feedback, such as customizing messages to users based on their baseline PA level, and user targeting, which adapts content based on user demographics (eg, gender and BMI [44]).

However, even personalized interventions can be insufficient, as they do not account for changes or natural fluctuations in users’ needs and preferences. This is a key issue for digital behavior change tools; personalization may only address who a user was in the past, rather than who they currently are, or may not meet a user’s needs in the moment [45]. *Adaptation* is a more advanced form of personalization, whereby application content is adjusted based on recent or immediate context [44]. This ensures that digital tools communicate information to users as it is currently relevant, rather than relying on data collected when the user adopts the tool initially. For example, a digital tool may account for a user’s recent daily steps when delivering feedback in an intervention for PA, so that the feedback is relevant to that day’s PA behavior. Digital platforms may be most effective when they are both personalized and adaptive [46,47]. As noted, there are individual differences and time-related and context-related variations in individuals’ preferences for and behavioral responses to social support and comparison, both generally [36,48,49] and specifically among women in midlife who have elevated CVD risk [32,50].

Effectively personalizing and adapting digital platforms require a detailed understanding of the target population. *UCD*, which encompasses several principles and methods aimed at deeply understanding intended end users [51], is widely used in industry settings (eg, product or service development) and is steadily gaining attention in academic research as the demand for engaging digital health platforms and services grows. UCD is iterative—new insights are used to drive and refine development throughout the process (eg, user testing informs design modifications). UCD methods have been used to develop digital health tools for many health behaviors and outcomes, including weight control and binge eating (via digital diaries and personas [52]), medication adherence (via interviews, needs assessments, and focus groups [53]), and chronic obstructive pulmonary disease (via background analysis, prototyping, and usability testing [54]). With respect to digital PA tools, UCD methods have been used to personalize apps for walking among breast cancer survivors (via needs assessment, prototyping, and usability testing [44]), sitting time reduction among office workers (via usability testing and user interviews [55]), and leisure time PA among adults living in close proximity to parks (via needs assessment and user evaluation [56]). Few existing studies, if any, have used UCD principles to develop resources for women in midlife, and no existing study has developed a digital PA tool specifically for this population.

### Aims of This Study

There is a clear need for more effective PA promotion resources for women in midlife, and digital tools are appealing because of their flexibility and ease of access. To address this need, our team of behavioral and computer scientists developed a personalized and adaptive web application that is tailored to the needs of this population. This tool is accessed via a web browser and allows users to self-select PA-related social support and social comparison opportunities, which are intended to bolster PA self-efficacy and motivation. Other behavior change techniques activated by the web application include PA self-monitoring, goal setting, and intention formation or planning [26,57]. Social support is provided via short, text-based messages, and comparisons are prompted via exposure to the profile of a peer. As described in the following sections, personalization occurs with respect to the user’s age and racial and ethnic identification, and the application adapts to the user’s level of PA behavior at each use. Of note, the web application is designed for brief, repeated use—specifically, 10 minutes per day for multiple consecutive days—on days when users wear a PA monitor (eg, pedometer or Fitbit). This allows for the assessment of variability in selections and responses, including subjective perception of selected content and objective PA behavior on a given day.

The overarching goal of this paper was to describe the UCD process of developing and conducting the initial evaluation of the web application prototype, including the results of feasibility, acceptability, and functionality testing with real end users from the target population of women in midlife with elevated CVD risk. We also note some of the lessons learned and present recommendations for future collaborations between behavioral and computer scientists to develop similar digital tools. This study was conducted in four stages: (1) initial application development, (2) feedback on the potential content of the web application (ie, content of peer profiles and messages) from the population of interest, (3) internal functionality testing, and (4) functionality testing with a different set of participants from the population of interest. Table 1 provides a summary of the goals, users, and methods for each stage.

**Table 1:** Summary of the 4 stages of web application development and testing.

Stage		Goal	Users	Methods
Initial development		Final storyboard and specifications to guide prototype programming	N/A^a^	Discussions between investigators, iterative storyboard generation, and feedback
Content feedback		Selection of profile images, formats, and messages in response to end users’ input	A total of 10 women in midlife, with ≥1 CVD^b^ risk conditions	In total, 5 qualitative interviews and 5 survey responses
**Internal testing**				
	Round 1	Identification of bugs or other problems to address with respect to web application functioning	A total of 9 behavioral science trainees (clinical psychology and health behavior) who were familiar with the web application	Totally, 9 sets of positive and negative testing
	Round 2	Identification of bugs or other problems to address with respect to full data collection procedure	A total of 3 behavioral science trainees (clinical psychology) who were not familiar with the web application	In total, 3 in vivo tests, each over 3 days (morning web application use, pedometer wear, and end-of-day survey)
Naturalistic functionality testing		Identification of bugs or other problems to address with respect to full data collection procedure and obtaining end-user feedback	A total of 5 women in midlife, with ≥1 CVD risk conditions	In total, 5 in vivo tests, each over 4 days (morning web application use, pedometer wear, and end-of-day survey)

^a^N/A: not applicable.

^b^CVD: cardiovascular disease.

## Methods

### Description of Web Application Content and Use Flow

Each user was provided a study identifier and unique link to the web application. Their researcher-created profile included their age and racial or ethnic identification, for personalization (see next paragraph); the web application does not collect any identifying information during use. The initial page of the application reminds users about the content they will encounter and instructions for optimal use of the application (eg, allocate approximately 10 minutes to engage with it via a web browser each morning). Then, the users are guided to report their step and active minute goals for the day from their PA monitors and reflect on their satisfaction with their PA behavior from the previous day.

**Figure 1:**
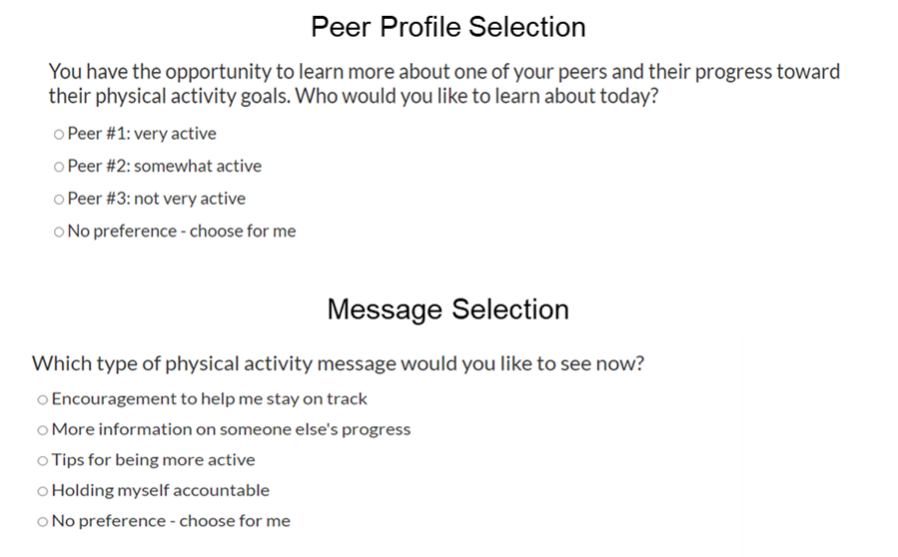
Web application screens that facilitate selection for peer profiles (social comparison targets) and messages (social support sources).

Next, social comparisons are prompted by exposure to a peer profile, which shows an image of a fictitious peer and brief text with this peer’s characteristics (ie, another woman who appears to be between 40 and 60 years of age, who is interested in increasing her PA; refer to Figure 1 for an example). Each profile text lists the peer’s average number of steps and active minutes per week, their favorite way to be physically active, their biggest challenge to being active, and their social context (ie, work situation, family, and caregiving responsibilities). Users can select one of four options: a highly active peer (upward target), a moderately active peer (lateral target), a not-so-active peer (downward target), or *no preference*. In the latter case, the system randomly selects an upward, lateral, or downward target with equal probability.

For the first peer shown at each daily use, personalization is achieved by showing images of peers whose age and racial or ethnic background align with those of the user; users who do not provide a racial or ethnic identification are indicated as race or ethnicity unspecified and view a peer image whose identification is randomly selected at each use. Each image is tagged with 2 relevant age ranges and 2 relevant racial or ethnic identifiers. Within these specifications, the application randomly selects an image and peer text from a database for each profile, ensuring that a user is not shown the same image or text on 2 consecutive profiles. Adaptation occurs by anchoring the peers’ average daily steps and active minutes to the user’s reported PA behavior from the previous day, and peers’ PA metrics are updated every day in response to changes in the user’s PA behavior. Specifically, highly active peers’ steps and active minutes are 130% of that of the user from the previous day, moderately active peers’ PA is 95% to 105% of that of the user from the previous day, and not-very-active peers’ PA is 68% of that of the user from the previous day. If PA behavior data are unavailable for the previous day, these adaptations are made using prespecified ranges. Users review the provided peer profile and then respond to a series of questions about their perceptions of the peer described.

Following peer profile reflections, social support is provided via exposure to a brief support message. Users are able to select one of four options for message category: encouragement, tips (ie, suggestions for ways to be more active and information about the benefits of activity for women), accountability (ie, suggestions for holding yourself accountable to your PA goals), or *no preference*. In the latter case, the system selects the user’s least-recently-used message category. Then, the system randomly selects encouragement, tips, or accountability message from a database (Figure 1). The system ensures that a user is not shown the same text on 2 consecutive messages. Users review the provided message and respond to a series of questions about their perceptions of the message, including how helpful they found it to be.

After completing these items, users are asked whether they would like to see a third message (yes or no). Those who select *no* are directed to the last set of questions for the day, which prompts PA intention formation via text entry. Those who select *yes* are offered a new set of options: encouragement, tips, accountability, another peer, or no preference. Users who attempt to select the same message category twice in the same use episode are prompted to *keep things interesting—select another type of message*, and the system does not allow them to move forward until they select a different category. Users who select to view a second peer for the day are not able to choose the type; they are randomized to a profile using the rules described previously for a *no preference* selection. After viewing the third message, the user responds to a series of questions about their perceptions and then is prompted to form PA intentions or plans via text entry, as described previously. Textbox 1 lists all the behavior change techniques activated by web application use.

**Physical activity (PA) behavior change techniques activated by the new web application.**

**Self-monitoring**

- Use of pedometer, which is worn on days of web application use
- Reflection on the previous day’s PA progress

**Goal setting**

- Identification of step and active minute goals for the day

**Social support**

- Selection of support message type to support PA motivation and behavior
- Reflection on message content and application

**Social comparison**

- Selection of comparison type (peer profile) to support PA motivation and behavior, personalized to match user’s age and racial or ethnic identification and based on user’s recent PA behavior (adapted to steps and active minutes)
- Reflection on response

**Planning or intention formation**

- Open-ended response to the following prompt: “Please describe your plan for reaching your activity goals today. How will you get your steps or active minutes in today?”

### Recruitment and Participants

#### Initial Application Development

PhD-level researchers in clinical health psychology (DA) and computer science (AFL) met regularly throughout 2019 and 2020 to develop the purpose, functions, and technical specifications of the web application (described in detail in the *Procedures* section). Both investigators were women; 50% (1/2) of them identified as White and 50% (1/2) identified as Latina.

#### Content Feedback From the Population of Interest

Women from the target population were recruited for 90-minute feedback interviews conducted via Zoom (Zoom Technologies, Inc). Eligibility criteria required that women be aged between 40 and 60 years (inclusive), with ≥1 risk conditions for CVD (ie, hypertension or prehypertension, prediabetes or type 2 diabetes, hyperlipidemia or hypercholesterolemia, metabolic syndrome, current smoker, or quit smoking in the past 3 months); that they were not currently pregnant; and that they had access to Zoom for the interview. Recruitment was conducted via electronic advertisements posted to the supporting institution’s announcement service and social media sites such as Twitter. Interviews were conducted in January 2021. Of the 6 women who were initially contacted about their interest, 5 (83%) enrolled and completed the interview. The average participant’s age was 47.4 (SD 6.66) years and BMI was 35.3 (SD 9.55) kg/m^2^. As all but one of these participants (4/5, 80%) identified as White, 5 additional participants were recruited to provide feedback via electronic survey, to ensure representation of perspectives of women from a range of backgrounds. For this group, the average participant’s age was 51 (SD 6.60) years and their BMI was 32.7 (SD 8.39) kg/m^2^. Of the 5 participants who completed the survey, 2 (40%) identified as Black, 2 (40%) identified as Latina, and 1 (20%) identified as East Asian.

#### Internal Functionality Testing

Investigators familiarized their team members (ie, not users from the target population) with the application’s purpose and previous stages of development. Then, the entire team conducted internal testing to confirm that the application functioned as intended. A total of 9 testers were included in the first round, of which 3 (33%) were doctoral students, 4 (44%) were undergraduate students in psychology, 1 (11%) was PhD-level program manager, and 1 (11%) was PhD-level investigator in clinical health psychology. All of them have backgrounds in digital health research. Of these 9 testers, 5 (56%) were women and 7 (78%) identified as White. In the second round, testers were 3 doctoral students in clinical psychology, without familiarity with the web application. All of them (3/3, 100%) were women aged 25 to 30 years; 67% (2/3) of them identified as White and 33% (1/3) identified as Black.

#### Feasibility, Acceptability, and Functionality Testing With the Population of Interest

Women were recruited using electronic advertisements posted to the supporting institution’s announcement service and social media sites (eg, Twitter), to engage in 4-day naturalistic functionality testing. In addition to the eligibility criteria described for content feedback, eligibility required that women did not have an active injury or illness that impeded their PA. A total of 11 women expressed interest in participating, and 5 (45%) women enrolled and completed these tests in June 2021. The average participants’ mean age was 50 (SD 6.26) years and BMI was 30.1 (SD 5.83) kg/m^2^. Of the 5 participants, 2 (40%) identified as Black, 1 (20%) identified as Latina, and 2 (40%) identified as White. None of these women (0/5, 0%) participated in the earlier phases of this study and thus, were naïve to the content of the web application. These users tested the application each morning for 4 consecutive days. They also wore either their personal PA monitors or a study-provided pedometer, completed end-of-day surveys on each day, and engaged in a brief exit interview at the end of data collection.

### Measures

#### Content Ratings (Initial Feedback)

Qualitative responses were noted for participants’ impressions of peers’ ages, racial or ethnic backgrounds, and professional circumstances for each image shown and for preferences regarding peer profile content. Participants who engaged in the initial interviews were asked to rate each PA message on a scale of 1 (*not at all*) to 10 (*extremely*), regarding (1) how much they liked each message and (2) how helpful they thought it would be for supporting their PA behavior. Participants were asked to briefly articulate their reasons for these ratings.

#### PA Monitors and End-of-Day Surveys (Second Phase of Internal Testing as Well as Feasibility and Naturalistic Functionality Testing)

Women who participated in the second phase of internal testing (3 trainees; 3 days each) and in feasibility/naturalistic functionality testing (5 women in midlife; 4 days each) wore a PA monitoring device during each test day. These participants used either their own personal device (eg, Fitbit or Apple Watch; worn on the wrist) or a study-provided Accusplit AX2720MV pedometer (worn on the waist or hip). These devices captured steps and active minutes (ie, high-intensity activity) throughout the day, and participants reported their totals from these devices at the start and end of each day. Participants reported their starting PA at the start of web application use each morning and daily totals in end-of-day surveys (sent via SMS text message or email).

#### Web Application Use and Technical Problems (Second Phase of Internal Testing as Well as Feasibility and Naturalistic Functionality Testing)

We determined the feasibility of web application use as intended by the percentage of times the application was accessed successfully and returned no errors (or other noteworthy problems), relative to the number of expected uses. Additional relevant data were related to devices and browsers used to access the web application, nature and replicability of reported errors, and application navigation information (eg, peer and message category selections).

#### Exit Interview (Feasibility/Acceptability/Naturalistic Functionality Testing Only)

Women in midlife who completed feasibility/naturalistic functionality testing engaged in a brief exit interview after the end of their tests, conducted via Zoom (15-30 minutes). Participants were asked about their overall experience with the web application, each component of the application, and whether they would be interested in future use. Given the complexity and lack of consensus regarding measuring acceptability in digital health research [58], responses in these domains were used to informally summarize participants’ views of acceptability (as described in the following sections).

### Procedures

#### Initial Development

Collaboration began in 2018, with support from internal and external funding provided to the first author (DA). Investigators met regularly in 2019 and 2020 to discuss the goals of a new tool, how it may be tailored to the needs of women in midlife with elevated CVD risk, and the process of identifying technical specifications. Although we considered a stand-alone mobile app as the outcome, we selected a web application owing to its versatility and ease of access across devices and platforms. During this time, the first author built on her previous studies with the population of interest [37,39,59] by conducting additional observational and qualitative studies [32,33,60,61] and collecting preliminary data to assess their needs and preferences. Insights from previous studies, discussions between investigators, and new participant responses informed the refinement of the goals of the new tool.

**Figure 2:**
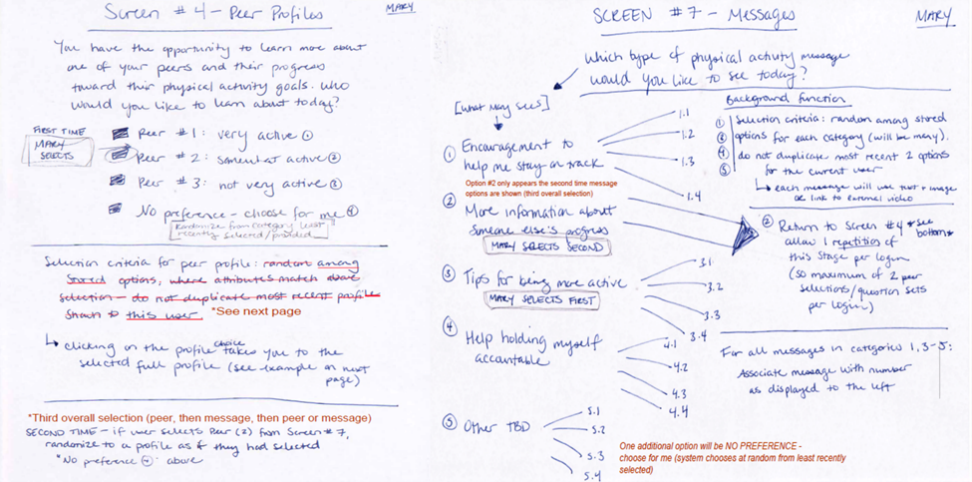
Pages from the final version of the web application storyboard, describing content and function for selection of peer profiles (social comparison targets) and messages (social support sources). Mary refers to the selections of a fictitious user, to indicate the expected flow.

In the spring and summer of 2020, investigators developed storyboards to specify the requirements of the web application. We developed a total of 7 storyboard drafts using an interactive process, which was led by the first author (DA) and modified with questions and input from the computer science expert (second author [AFL]). Figure 2 shows screenshots of the final storyboard, illustrating some of the important steps of the user experience with the web application. This resource included 37 sheets describing the intended progress through 15 pages of the application, including descriptions of progress for 2 fictional users and skeleton of a database that would house participants’ selections and responses as they used the application. The storyboarding process led to the creation of a penultimate set of technical specifications, which the computer scientist (AFL) used to develop the code for the initial draft of the application.

Development of the application occurred during the summer and fall of 2020. We chose the Django framework for its integration of front-end and back-end development and its secure superuser view that allows team members to edit the database entries for peer texts and support messages. The application was deployed on an Amazon Web Services ec2 instance using the SQLite database. During this time, the first author (DA) and trainees in behavioral science created a preliminary set of peer profiles and support messages. This included images, profile text, and message text. Images of women who appeared to be aged between 40 and 60 years were selected from open-access web-based databases [62] to represent a range of racial or ethnic and professional backgrounds. Profile text was generated based on the investigators’ knowledge of and experience with prompting PA-based social comparisons, creating 5 versions of the same 2 profiles that offered different levels of detail. Finally, 18 support messages (6 each for encouragement, tips, and accountability) were generated based on those used in previous studies [63-65], with modifications to address the needs of women in midlife and attention to the possibility of limited access to fitness facilities during the COVID-19 pandemic (eg, exercises that are easy to do at home, without equipment).

#### Content Feedback With the Population of Interest

**Figure 3:**
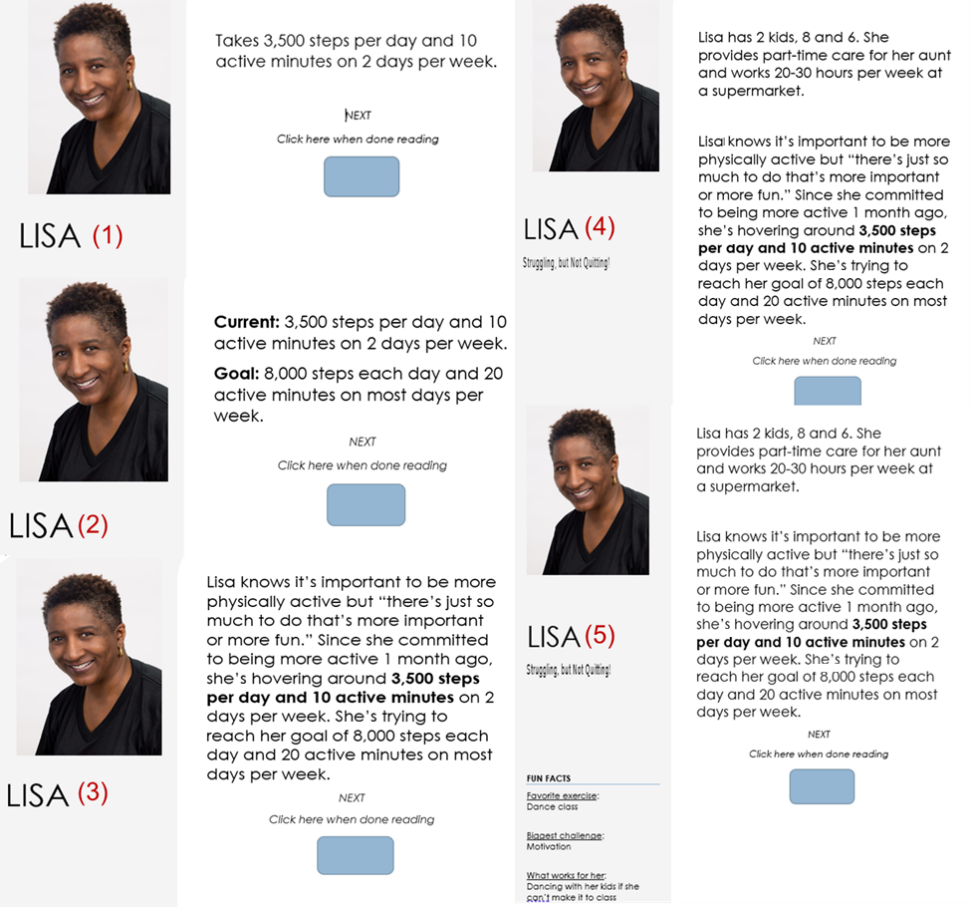
Versions of possible peer profiles rated by end users during content feedback stage.

Procedures were approved by the supporting institutional review boards (IRBs), and the participants provided written informed consent via Adobe electronic signature (5/10, 50%). Participants met with the first author (DA) for individual, 90-minute interviews via Zoom. These interviews focused on gathering women’s perceptions, preferences, and suggestions for improvements to sample peer images, profile text, and messages. After initial overview of the session and introduction to the purpose of the web application, participants were asked to view a series of peer images and indicate their perceptions of the woman’s age, racial or ethnic identification, and profession. Then, they viewed 5 versions of each of the 2 peer profiles and were asked to verbally evaluate each version (ie, strong vs weak preference for each, relative to the previous version, and their reasons). These profiles showed minimal information initially and increased in the number of facts presented in each version (Figure 3). Next, participants were asked to read each of the 18 messages and rate each one on the scales described previously. Finally, they were asked to rate their interest in using the web application to support their PA efforts in the future on a scale of 1 (not at all interested) to 5 (extremely interested). Women who provided feedback via electronic survey (5/10, 50%) provided informed consent by clicking a button to verify that they consented to proceeding with the survey; they were given a brief explanation of the web application and asked to respond to a subset of the stimuli described previously.

The interviewer (first author [DA]) took detailed notes during the Zoom feedback sessions to capture numeric ratings, narrative explanations for these ratings, and other suggestions from participants. Then, the interviewer and another member of the behavioral science team reviewed and synthesized these notes and responses from the participants who completed the survey. They identified common themes and recommendations for changes, which were implemented as described in the following sections. A third member of the behavioral science team was consulted for input on messages that received a wide range of responses, and disagreements about changes were resolved by consensus.

#### Internal Functionality Testing

In the first phase, research team members were randomly assigned to user accounts representing women in the target age range, from varying racial or ethnic backgrounds, and asked to conduct a series of tests of the web application. First, they completed a round of positive testing (using valid data entry) to verify that the application worked as expected. Then, they completed a round of negative testing (using invalid or improper data entry) to check for unexpected conditions. Each user conducted between 5 and 20 tests over the course of 4 months, with application updates occurring between each round of tests (February 2021 to May 2021). A subset of team members (5/9, 56%) also exported data files for recent use episodes to check for errors in recording the use variables in the database.

Users in the second phase of testing received instructions via email for testing the application between 8 AM and 9 AM each day for 3 consecutive days, wearing their personal PA monitors, and completing end-of-day surveys on each day. Of the 3 users, 1 (33%) user tested as a woman with racial or ethnic identification the same as her own (Black), and 2 (67%) tested as women not matched to their racial or ethnic identification (the White trainee users tested as a Latina woman and as a woman whose identification was unspecified, respectively). End-of-day surveys were sent via SMS text messages at 9 PM each night and were completed via mobile device by 9:45 PM. Before users accessed the web application on the following day, members of the research team entered their step and active minute totals to the application, to enable adaptation of the PA levels shown in peer profiles. The investigators were available during the application test window each morning (8 AM to 9 AM) to receive and address reports of errors and access difficulties.

#### Feasibility, Acceptability, and Functionality Testing With the Population of Interest

All procedures were approved by the relevant IRBs, and participants provided written informed consent via Adobe electronic signature (5/5, 100%). Each participant met with the first author (DA) via Zoom for introduction to the web application and study procedures, during which the first author used screen share to show participants an episode of web application use in real time. Participants who requested to use a study pedometer (3/5, 60%) received these devices and printed instructions in the mail before this meeting (with prepaid postage for the return mailing) and were invited to ask questions about intended use for data collection. Next, participants received an overview of the end-of-day survey content and procedures and engaged in a live test of receiving surveys via SMS text messages. The interviewer collected information about participants’ wake and sleep times and discussed their preferred time for using the application and receiving end-of-day surveys, according to their wake and sleep times. Specifically, participants were asked to use the web application within 2 hours of waking up and to complete the end-of-day survey within 1 hour before going to sleep. The investigators were available during the application test windows each morning to receive and address any reports of errors or access difficulties. Finally, before participants accessed the web application on the following day, the first author entered the step and active minute totals to the application, to enable adaptation of the PA levels shown in peer profiles. Following the procedures outlined previously, the interviewer (first author) took detailed notes during the Zoom exit interviews to capture participants’ views of acceptability. The interviewer and another member of the behavioral science team reviewed and synthesized these notes to generate a summary of the acceptability feedback.

### Ethics Approval

The IRB at Rowan University approved this study (PRO-2020-163).

## Results

### Development

Refinement of the initial idea for the web application occurred over a period of 1 year. This period involved regular discussions among investigators, storyboarding, identifying technical specifications, and designing a database. Investigators created 7 drafts of the web application storyboard using a process of iterative feedback. At each stage, discussions generated questions to be answered and functions to be refined, which would allow for individual user personalization and day-to-day adaptation. The initial version of the storyboard used 8 pages to describe the 9 distinct screens needed to achieve a full-use episode. The final version used 37 pages to specify back-end decision points, 15 distinct screens needed to achieve a full-use episode, and full examples of 2 different users’ experiences of a full-use episode. Separate files illustrated the database (where users’ selections, entries, time spent in using the application, and end-of-day data are collected and stored), administration pages (for creating users, entering end-of-day data, and downloading database files), and a penultimate set of technical specifications. The software implementation, deployment, testing, and support tasks required approximately 3 months of effort from the computer science expert (second author [AFL]). Finally, we deployed and tested the web application as described previously.

### Content Feedback

Gathering feedback on potential web application content occurred during the later stages of programming, over a 4-week period. Participants who engaged in 90-minute interviews via Zoom (5/10, 50%) reported strong overall interest in using the web tool under development to support their PA efforts (mean score 4.5 out of 5). Regarding peer images, there was high agreement regarding perceived age (87%), racial or ethnic background (88%), and types of careers (85%). A total of 2 images were perceived as depicting women aged between 40 and 46 years, 3 images as women aged between 45 and 52 years, and 2 images as women aged between 52 and 60 years. A total of 3 images were perceived as depicting Black women (or multiracial with Black as an identification), 4 as Latina women (or multiethnic with Latina as an identification), 2 as White women (or multiracial with White as an identification), and 1 as Middle Eastern or South Asian (Indian or multiracial with one of these as an identification). A total of 3 images were described as portraying women with administratively focused jobs (eg, office assistants and business managers), 2 as teachers, and 3 as other (eg, coaches and retired). All images were deemed acceptable for the stated purpose, and participants indicated that some variety across images (eg, in types of clothing) may make the peer profiles relatable to a wide range of women in midlife.

A total of 2 images were perceived as *intimidating* by ≥1 participant. The first image was described as projecting a “no nonsense” attitude (however, still moderately welcoming), whereas the second appeared to “have it all” (ie, fit, affluent, and carefree). These happened to be the 2 images used for the next set of prompts related to the amount and types of information that participants perceived as useful for inclusion in peer profiles. For this set of prompts, the participants viewed 5 different versions of each woman’s profile, each increasing the amount of information presented. For both profiles, participants indicated that the middle version provided the optimal amount of information (ie, version 3 in Figure 3). Although 60% (3/5) of the participants specifically expressed interest in the addition of *fun facts* in each profile (as these made the profiled women seem more *relatable*), they indicated that the short versions were the best for providing useful information efficiently.

Participants’ overall recommendations for PA messages were to keep them short; however, there was considerable heterogeneity in the subjective response to each message. Consider the following message: “We’re often told how important physical activity is to our health. But why is it important to you? Remind yourself of YOUR reasons to be more active.” This message received a score of 0 from one participant (regarding how much she liked the message and found it helpful); she indicated that the message implied that PA “must not be important if I’m not doing it,” which was not motivating. However, the same message received a score of 10 from another participant (who said it was “thought-provoking and affirming”), and the average ratings for liking and helpfulness were 6.2.

Examples of the highest-rated and lowest-rated messages are listed in Table 2. The lowest-rated messages were removed or substantially revised; all other messages received minor adjustments to wording or formatting (eg, whether parts of the text were underlined or bulleted), based on participant feedback.

**Table 2:** Sample messages and ratings and comments by end users, obtained during the content feedback stage.

Messages	Ratings (1-10 scale)			Comments			
	Liking, mean (SD); range	Helpfulness, mean (SD); range					
Do you schedule meetings and appointments on your calendar, and usually show up to them? Could you do the same for physical activity? Make it easy – add time to be active on your calendar, and make it a point to show up like you would with any other obligation!	4.1 (1.67); 3.5-7	4.3 (2.11); 3.5-7	“I agree that this could work but it causes me stress - I have too many appointments already.” “This is a good suggestion, but reword it so that it’s not asking so many questions.”				
Do you have responsibilities such as childcare, or taking care of others you care about? Lack of time or energy due to these responsibilities can make physical activity difficult, but it doesn’t mean that you can’t be more physically active. Try going for walks, light stretching, or standing while watching TV. You can do all this with your loved ones, or try it during short windows when you have a moment to yourself.	5.4 (2.97); 2-10	5.3 (1.99); 2-7	“This one is really long and it’s not personal - seems too general.” “Too long but it’s a good reminder.”				
You don’t have to work on your physical activity goals alone! Support from family and friends can help you to achieve your goals. Let someone close to you know about your goals, so they can provide you with support and help hold you to them!	8 (2.12); 5-10	7.8 (1.92); 5-10	“This is good advice. It helps hold you accountable to tell someone else.” “If I were going to do this I would have done it already.”				
When setting physical activity goals each week, try to make them clear and measurable. Instead of a goal of “walking more,” try: walking 3 times this week for 10 minutes each time. This will help you stay on task and make it easier to know when you’ve met your goal. Then you get a sense of accomplishment when you know you’ve met your goal!	8.3 (1.20); 7-10	8.1 (1.34); 7-10	“I like this a lot. It’s clear and actionable.” “I agree! Great suggestion.”				

### Internal Testing

#### Round 1

Of the 25 positive tests conducted, 2 (8%) resulted in initial errors, and they were resolved. A further 16% (4/25) of the tests revealed bugs, including failing to offer a second message and repeating the same content twice in 1 use episode. Both of these problems were resolved after the conclusion of round 1 testing. Negative testing (involving 20 use episodes) identified two application navigation pitfalls: using the back button allowed for many selections in the same use episode and entering spaces or other non–language-based text was accepted as valid responses to the open-ended questions. Across both types of tests, several team members also indicated that certain details of peer profiles did not match their intended ages. For example, a subset of photos of women in their late fifties was paired with profiles describing them as having very young children.

At the end of round 1, the investigators consulted and agreed that the application should insist that the user proceed strictly in the order described previously, irrespective of the user’s attempts to use their browser’s back button. The second author (AFL) made system modifications such that the user is required to complete a day’s survey on a single device and browser, and the user’s attempt to use their browser’s back button redirects them to the next screen. These updates were completed before the functionality testing with end users. The application specifications did not include procedures for preventing nonlanguage text responses to the open-ended questions. In contrast, the peer profiles were intended to be tagged with age indicators (similar to peer images), but this step was skipped in the build process. As the latter two issues were deemed substantial revisions, they were noted and postponed until a future round of substantive updates.

#### Round 2

Across 3 testers and 3 days of use episodes (total of 9 episodes), 11% (1/9) of the test uses of the web application resulted in an error. The error for the assigned client could not be resolved and the tester was assigned a new client ID. After this substitution, all use episodes were successful, and all end-of-day surveys were completed as intended. Of the 3 testers, 1 (33%) tester noted that she received the same message twice in the same use episode, despite choosing different message categories, and this error was logged for correction. All testers (3/3, 100%) expressed their perception that the web application would be useful for supporting women’s PA efforts and indicated that the web application’s personalization and adaptation features were effective for tailoring their experience of peer profiles. Testers also offered additional feedback on the wording of peer profiles, messages, and end-of-day surveys to clarify the specific experiences of interest (eg, a missing text box for entering details if the *other* response to a multiple-choice item was selected).

### Feasibility, Acceptability, and Naturalistic Functionality Testing

Testing with a different group of women from the target population was conducted over 4 weeks. All the participants (5/5, 100%) were able to access the web application on their personal devices. This included the use of desktop and laptop computers, tablets, and smartphones, across Chrome and Safari browsers. A total of 60% (3/5) of the participants did not encounter any errors or problems with access on any of their days of participation. Of the 5 participants, 1 (20%) participant received an error after starting use (ie, getting to the starting page without entering any data), closing her browser, and attempting to restart later. As the application allows only 1 use per client per day, this difficulty demonstrated correct functionality. The participant was able to access the application on the same day after her first attempt was deleted by the research staff. The final participant received error messages for attempts to access the web application on her smartphone but encountered no problems when she switched to her laptop computer the following day. The initial problem could not be duplicated by the research staff but was noted in case of repeated difficulties. Thus, of the 20 expected use episodes (5 participants; 4 days each), 18 (90%) were completed and resulted in use data without incident and the remaining 2 (10%) were missing, owing to access difficulties described previously. In total, 85% (17/20) of the episodes were informed by previous end-of-day step and active minute totals.

Participants’ peer profile and message selections are summarized in Table 3. Across participants and use episodes, initial peer type selections were most frequently lateral or downward targets (6/18, 33% each). Upward targets and *no preference—choose for me* were selected during 17% (3/18) of episodes each, by a different participant each time; *no preference* was selected on different days of use each time, whereas 67% (2/3) of the selections of upward targets were on the last days of use. All of the participants (5/5, 100%) selected at least two different peer options. Of the peers actually displayed, lateral or downward targets were seen in 39% (7/18) of episodes each, and upward targets were seen in 22% (4/18) of episodes. As noted, these directions were relative to participant’s PA behavior on the previous day. Initial message type selections were predominantly those providing accountability (7/18, 39%), followed by encouragement (6/18, 33%) and tips (3/18, 17%). *No preference—choose for me* was selected only twice, by a different participant and on a different day of observation each time. A second peer and an encouragement message were chosen twice each; a tip message and *no preference* were selected once.

**Table 3:** Summary of peer profile and message selection types during end-user functionality testing stage (5 users; 4 days each).

Categories	Selections of episodes (n=18), n (%)
Upward peer	3 (17)
Lateral peer	6 (33)
Downward peer	6 (33)
No preference for peer	3 (17)
Encouragement message	6 (33)
Tips message	3 (17)
Accountability message	7 (39)
No preference for message	2 (11)

During the exit interviews, all participants (5/5, 100%) indicated that their overall experience with the web application was positive; 80% (4/5) of them indicated that they found the application very helpful in setting a positive tone for their PA for the day and reminding them to be more active. For example, a participant stated that she noticed herself walking her dog more often as a way to increase her PA. The final participant reported that she found the web application acceptable. As she was previously very active and used several of the application’s suggested approaches at that time, the content was “not new” to her. However, she reported an expectation that it would be useful for someone just starting an effort to be more active. All participants (5/5, 100%) indicated that they would be interested in using an expanded version of the web application to support their ongoing PA efforts, with 60% (3/5) of them specifying that use would be most beneficial in conjunction with PA coaching (ie, additional, formal guidance to build behavioral and psychological skills).

## Discussion

### Principal Findings

Women in midlife—particularly those who have health conditions that exacerbate their increasing risk for CVD—would benefit from PA-specific resources that address their social preferences and needs. This paper describes a multidisciplinary, iterative, UCD approach for developing a personalized, adaptive web application for this purpose. Our approach involved four stages: initial design and building, gathering content feedback, internal testing, and naturalistic functionality testing with end users. Changes were made to the application’s content, function, and navigation in response to feedback at each stage, which resulted in a prototype that showed evidence of feasibility and acceptability among the target population.

Our initial findings also suggest the possibility of heterogeneity in the PA preferences and experiences of women in midlife. Some social content was consistently rated as favorable, whereas other content generated wide ranges of favorability, and women showed noteworthy variability in their support message and peer (comparison target) selections in their daily lives. Some of this variability may be due to curiosity and desire to explore the available options [66,67]. The content that women in midlife want or believe will be most helpful also likely varies from day to day (eg, with mood and other contextual factors). An important next step is to conduct studies with long test periods and large samples to determine the extent of stability versus ongoing variability in women’s selections and responses. Incorporating UCD principles and methods throughout the development process provided insight into the needs and preferences of our target population. By taking an iterative approach, we identified issues early, which allowed for refinement before further testing. This process was made easy by regular, planned discussions between the behavioral science and computer science teams and ensured a shared vision and scope for the project.

### Uses and Future Directions for a New PA Web Tool

There are multiple potential uses for our new web application, and our future studies will investigate the utility of the tool for each purpose. First, as suggested by members of the target population, this tool may be used as an adjunct to and extension of more traditional PA coaching. Individual or group-based coaching, which often occurs via live interaction (in person or through phone calls or video calls), can provide a range of psychological and behavioral skills for adopting or maintaining regular PA routines (eg, intention formation and planning [68]). The web application, available daily, can supplement these interactions by providing content that reinforces skills and guides actions on demand in women’s daily lives. Similarly, the web application can serve as a low-intensity introduction to some basic PA skills (eg, goal setting) and help women to determine the types of support or behaviors that may work best for their goals. Given its low intensity, the web application is unlikely to be widely effective as a stand-alone intervention for PA adoption. However, there may be a subset of women in midlife who benefit from the minimal, noninvasive, personalized, and adaptive support that it offers, either for initial PA adoption or for reinitiation of previous PA routines (eg, as women recover from PA changes owing to the COVID-19 pandemic [69,70]).

We also see potential for more innovative uses of the web application, beyond traditional PA support. For example, as noted, what users want and what works for prompting behavior change in daily life do not always align [41]. When combined with objective PA monitoring, data from the new tool can facilitate the assessment of alignment between a user’s social PA content preference (selection), their subjective response to this content (liking or perceived helpfulness), and their daily PA behavior. This can be informative in 2 ways. First, from a basic science perspective, such assessment on a large scale will indicate the prevalence and distinct types of misalignments. This can inform our understanding of phenomena such as cognitive biases that interfere with PA, including strong preferences for and liking of content that is self-enhancing (eg, downward comparison targets [71]), despite the behavioral benefit of exposure to less-enhancing contact, such as upward comparison targets [72]. Second, from an intervention perspective, information about any observed misalignment of preferences and behavioral responses can be offered as feedback (eg, from PA coaches). This can enable women to gain insight into patterns that facilitate or inhibit their PA behavior change.

A final potential use of the new web application is for assessing the alignment of preferences and responses between users, which also has implications for PA interventions. Specifically, the procedures described in the naturalistic functionality testing phase can be used to generate a profile or type for each user. Content selections and both subjective and behavioral responses can determine the *optimal* social support and comparison exposures for promoting a user’s engagement with the tool and PA behavior [45]. At the same time, responses to survey items and PA behavior data can determine the type or types of support and comparison targets that the user would offer to other women in midlife. Then, this profile of optimal social input and likely output can be matched with a complementary profile to create PA partner dyads that are likely to meet each other’s social needs. This process can maximize the power of the social environment of PA interventions to promote PA among women in midlife.

The present set of studies represents only the formative steps in a long process to fully develop and test a new digital health tool. Consistent with many of the existing UCD studies, we used both quantitative and qualitative methods with modest sample sizes [73]. Each of these potential uses of the web application will require the noted substantive revisions to the tool (which are currently underway) and large-scale testing with large and more diverse samples of women in midlife.

### Lessons Learned and Recommendations for Future Digital Health Collaborations

The present series of studies and the creation of a new digital health tool resulted from close collaboration between behavioral and computer scientists. This type of collaboration is becoming more common and desirable to ensure that the development of a tool to support health behavior change is informed by a range of relevant expertise [74]. Such partnerships can be extremely fruitful but are not without challenges. For example, the fields of behavioral and computer science prioritize different methods and often share little common language. In this study, although we developed the storyboard and specifications for the web application over several months and a series of iterations, potentially important navigation rules were left unspecified (ie, use of the web browser’s back button, entry of spaces, or nonlanguage text as open-ended responses). These and similar oversights may be owing to behavioral scientists’ implicit assumptions and lack of familiarity with the problems that such errors can cause, and thus, failing to specify all desired rules ahead of time. To avoid the need for major changes after the initial tool is built—particularly if funding for such changes is limited or uncertain—there is additional pressure on computer science team members to foresee all types of possible pitfalls. In an academic setting, where funding for the development of digital tools may be scarce, early discussions about how and when substantive, unforeseen changes will be handled are critical.

Behavioral scientists also tend to focus, from the beginning, on a process of human subjects research that involves regulatory oversight, informed consent discussions, certain types of documentation (eg, test user feedback), and multiple attempts to secure additional (limited) funding. This process is intentionally methodical and can be quite slow as a result [74]. Computer scientists may not be familiar with the IRB processes, training required to participate in studies with human participants, and delays that these may cause. Computer scientists may prefer agile and iterative software development methods with multiple rounds of user feedback, but may not realize, at the beginning, the time and effort required to obtain IRB approvals and recruit participants. For this collaboration, although we were aware of the potential regulatory challenges from the beginning, we encountered difficulties regarding questions about permissions for server housing and IRB questions about the collection of protected health information via the new web application. The web application was designed to protect users’ identities and does not collect protected health information; however, the process of effectively communicating this to the IRB and securing permission to house the web application on a commercial server was lengthy and involved. This was one of the several delays in the large process of web application development and testing that contributed to the extension of the project time line. The onset of the COVID-19 pandemic was the cause of another delay, as part of the investigators’ research time was redirected to other professional and personal responsibilities [75].

### Strengths, Limitations, and Conclusions

Despite these delays, this study had several strengths, including a base of foundational work with the population of interest [32,37,60,61,69], adherence to widely recommended UCD principles [51,73], emphasis on recruiting women from racial or ethnic minority backgrounds to maximize diversity in small samples, and collaboration between flexible researchers who are committed to the project. This process also benefited from multiple phases of internal testing, including a range of testers who were familiar with and those who were naïve to the web application specifications and phases of both positive and negative testing. There were also noteworthy limitations to our study. For example, all data collection processes and feedback sessions with women in midlife were conducted by the first author (DA), who is the principal investigator of the project. This was intentional to ensure consistency in interview style and optimal synthesis of findings across study stages, particularly for the small samples recruited and the resources available. However, knowing that they were speaking to the project director may have heightened the participants’ social desirability or self-presentation behaviors. The first author’s direct involvement can also enable confirmation bias regarding the conclusions drawn (eg, from interview feedback). Interview scripts were designed to probe dislikes or nonpreferred content to limit this concern, and additional team members were consulted to provide an additional perspective, but it remains a possibility. This may be particularly relevant for the assessment of acceptability, which was performed informally in this study. In future studies, it would be ideal to record interviews and have multiple raters use a standard set of guidelines [76].

However, it is important to note that these procedures may not be possible at formative study stages owing to limited financial support to protect team members’ time for such activities. Relative to the resources available for later stages of the process, such as efficacy testing for an existing digital health tool, research funding for the formative stages of tool development is often much more modest. Consequently, creative thinking, patience, and persistence are critical to the early success of such a venture. Together, this series of studies provides a useful example of how to approach these formative stages to gain necessary insights from the population of interest and to identify pitfalls and the next steps for future studies.

## Abbreviations

CVD: cardiovascular disease
IRB: institutional review board
PA: physical activity
UCD: user-centered design
